# The Oral-Brain Axis: Mechanistic Insights Linking Periodontitis With Alzheimer’s and Parkinson’s Diseases

**DOI:** 10.7759/cureus.111856

**Published:** 2026-06-30

**Authors:** Roopse Singh, Shilpi Asthana, Anveksha Arya, Mayur Kaushik, Mehvish Khan

**Affiliations:** 1 Department of Periodontology and Implantology, Subharti Dental College and Hospital, Swami Vivekanand Subharti University, Meerut, IND

**Keywords:** alzheimer’s disease, blood-brain barrier, neurodegenerative diseases, neuroinflammation, oral-brain axis, oral microbiome, parkinson’s disease, periodontal disease (pd), periodontitis

## Abstract

Neurodegenerative diseases, including Alzheimer’s disease (AD) and Parkinson’s disease (PD), are major causes of disability and mortality worldwide. Emerging evidence suggests that chronic peripheral inflammation and microbial dysbiosis may contribute to neurodegenerative processes. The oral-brain axis has recently gained attention as a biological framework linking oral microbial communities, systemic inflammatory responses, immune regulation, and central nervous system function. Within this context, periodontitis, a prevalent chronic inflammatory disease driven by oral dysbiosis, has been proposed as a potential modifiable risk factor for neurodegeneration.

This narrative review examines current evidence supporting the oral-brain axis and its role in the relationship between periodontitis and neurodegenerative disorders. Key mechanisms include systemic dissemination of periodontal pathogens and their virulence factors, persistent inflammatory signaling, blood-brain barrier dysfunction, neuroimmune activation, oxidative stress, and protein aggregation. Particular attention is given to the contribution of *Porphyromonas gingivalis* and associated virulence factors to neuroinflammation, amyloidogenesis, and neuronal injury. Epidemiological, clinical, and experimental studies linking periodontal disease with cognitive decline, Alzheimer’s disease, and Parkinson’s disease are also discussed. Current evidence supports a biologically plausible association between periodontal disease and neurodegeneration through interconnected microbial, inflammatory, and vascular pathways. Although causality remains to be established, the oral-brain axis provides valuable insight into potential mechanisms underlying this relationship. Improved understanding of these interactions may facilitate the development of preventive and therapeutic strategies that integrate oral healthcare with approaches aimed at preserving neurological health and reducing the burden of neurodegenerative diseases.

## Introduction and background

Neurodegenerative diseases are a major global health challenge and a leading cause of disability and mortality worldwide. Alzheimer’s disease (AD) and Parkinson’s disease (PD) are the most prevalent disorders, and their burden is expected to increase with population aging. Despite advances in understanding their pathogenesis, current therapies remain largely symptomatic, emphasizing the need to identify modifiable risk factors and preventive strategies [1].

Growing evidence indicates that chronic inflammation and immune dysregulation contribute to neurodegenerative disease progression. Persistent neuroinflammation, characterized by activation of microglia and astrocytes, promotes neuronal injury through the release of pro-inflammatory cytokines, reactive oxygen species (ROS), and other neurotoxic mediators. Peripheral inflammatory conditions may further exacerbate these processes by disrupting the integrity of the blood-brain barrier (BBB) and amplifying systemic immune signaling [2].

The oral-brain axis has emerged as a conceptual framework describing the bidirectional interactions between oral microbial communities, systemic inflammation, immune responses, and central nervous system function. Oral dysbiosis, particularly periodontitis, has gained attention as a potential contributor to neurodegeneration because periodontal pathogens and their virulence factors can disseminate systemically and induce chronic inflammatory responses [3,4]. Periodontitis is associated with elevated levels of circulating inflammatory mediators, including tumor necrosis factor-alpha (TNF-α), interleukin (IL)-1β, IL-6, and C-reactive protein (CRP), which may influence neurological health [5].

Experimental studies have detected periodontal pathogens and their virulence factors in brain tissues and demonstrated that chronic oral infection can promote neuroinflammation, amyloid-beta accumulation, tau pathology, and cognitive impairment, providing biological plausibility for the oral-brain axis [6,7]. Epidemiological studies have further reported associations between periodontitis and cognitive decline, dementia, AD, and PD, although causal relationships remain unestablished [8-10].

Given the increasing recognition of oral health as a determinant of systemic and neurological well-being, this narrative review examines current evidence linking periodontitis with neurodegenerative diseases through microbial, inflammatory, immune, and vascular mechanisms, with particular emphasis on oral dysbiosis, pathogen dissemination, neuroinflammation, BBB dysfunction, and their implications for disease prevention and therapeutic intervention.

Method

Review Design

This article was conducted as a narrative review to provide a comprehensive overview of the current evidence regarding the oral-brain axis and its potential role in linking periodontitis with Alzheimer’s disease (AD) and Parkinson’s disease (PD). The objective was to critically summarize and integrate findings from clinical, epidemiological, experimental, and mechanistic studies rather than to perform a systematic review or meta-analysis.

Literature Search

A comprehensive literature search was performed to identify relevant publications investigating the association between periodontitis, the oral microbiome, and neurodegenerative diseases. Electronic databases including PubMed/MEDLINE, Scopus, Web of Science, Embase, and Google Scholar were searched from database inception through March 2026.

Searches were conducted using combinations of keywords and Boolean operators, including “oral-brain axis,” “periodontitis,” “periodontal disease,” “oral microbiome,” “oral health,” “gingivitis,” “tooth loss,” “neurodegeneration,” “Alzheimer’s disease,” “Parkinson’s disease,” “dementia,” “mild cognitive impairment,” “cognitive impairment,” “neuroinflammation,” “blood-brain barrier,” “*Porphyromonas gingivalis*,” “gingipains,” “lipopolysaccharide (LPS),” “amyloid-beta,” “tau,” and “systemic inflammation.” The reference lists of relevant articles and recent review papers were also manually examined to identify additional publications.

Study Selection

Relevant peer-reviewed articles published in English were selected based on their relevance to the objectives of this review. Priority was given to original research articles, clinical investigations, cohort and case-control studies, systematic reviews, meta-analyses, and experimental studies that examined the association between periodontal disease, oral microbial dysbiosis, neuroinflammatory mechanisms, and neurodegenerative disorders. Editorials, conference abstracts, opinion articles, and studies not directly related to the oral-brain axis were not considered.

Narrative Synthesis

The selected literature was critically reviewed and synthesized narratively. Evidence from human clinical studies, epidemiological investigations, animal models, and in vitro experiments was examined and organized according to major themes, including periodontal dysbiosis, microbial translocation, systemic inflammation, blood-brain barrier dysfunction, neuroinflammation, and disease-specific mechanisms in Alzheimer’s disease and Parkinson’s disease. Given the heterogeneity of study designs, populations, outcome measures, and experimental methodologies, a quantitative synthesis or meta-analysis was not appropriate. Therefore, findings are presented as a qualitative narrative synthesis to provide an integrated overview of the current evidence, identify emerging mechanistic insights, and highlight areas requiring further investigation.

## Review

The oral-brain axis: a conceptual framework

The oral-brain axis is an emerging biological framework describing the bidirectional communication network linking oral microbial communities, systemic inflammatory pathways, host immune responses, and central nervous system (CNS) function. While the gut-brain axis has been extensively studied, growing evidence indicates that the oral cavity represents an additional and clinically relevant source of microbial and inflammatory signals capable of influencing neurological health.

The oral cavity harbors one of the most diverse microbial ecosystems in the human body, consisting of more than 700 bacterial species together with fungi, viruses, and archaea [11]. Under physiological conditions, these microorganisms coexist in a balanced symbiotic relationship with host tissues. However, disruption of microbial homeostasis results in dysbiosis, favoring the proliferation of pathogenic species that initiate chronic inflammatory responses and facilitate systemic dissemination of microbial products [12].

The oral-brain axis encompasses several interconnected pathways. First, oral pathogens and their virulence factors may enter the systemic circulation through ulcerated periodontal tissues, resulting in transient bacteremia and dissemination to distant organs [13]. Second, chronic periodontal inflammation promotes sustained release of pro-inflammatory cytokines and acute-phase reactants capable of influencing CNS homeostasis. Third, systemic inflammatory mediators and bacterial products may compromise blood-brain barrier integrity, facilitating access of peripheral inflammatory molecules to neural tissues. Finally, direct microbial invasion through hematogenous routes or cranial nerves has been proposed as an additional pathway contributing to neuroinflammation and neuronal injury [4].

**Figure 1:**
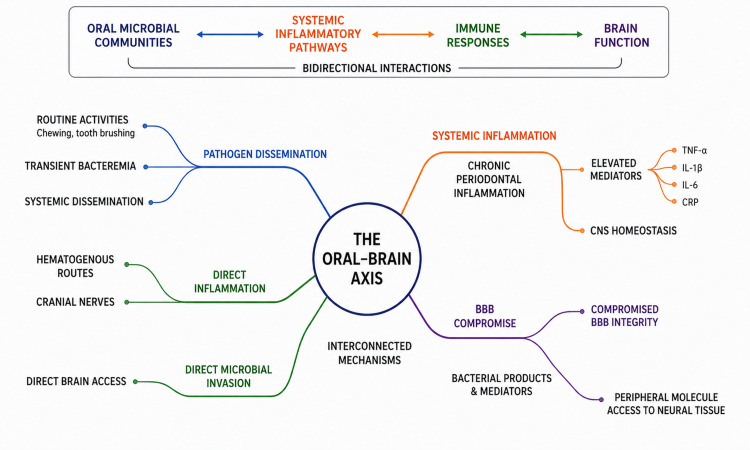
Understanding the oral-brain axis framework. Illustration of the bidirectional interactions between oral microbial communities, systemic inflammatory pathways, immune responses, blood-brain barrier (BBB) integrity, and brain function. The framework highlights key mechanisms, including pathogen dissemination, chronic systemic inflammation, direct microbial invasion, and BBB dysfunction, through which oral dysbiosis may influence neurological health and neurodegenerative processes. This image was created by the authors of this study using Microsoft PowerPoint 2021 software (Redmond, WA: Microsoft Corp.).

Importantly, communication within the oral-brain axis appears to be bidirectional. Neurodegenerative diseases frequently impair motor coordination, cognition, and self-care abilities, leading to compromised oral hygiene and increased susceptibility to periodontal disease. Consequently, oral and neurological health may influence one another through a complex network of microbial, inflammatory, vascular, and immunological interactions. Understanding these interconnected mechanisms provides a foundation for investigating the role of oral health in neurodegenerative disease pathogenesis. The principal components and interconnected pathways of the oral-brain axis are summarized in Figure 1.

Periodontitis as a systemic inflammatory burden

Periodontitis is a chronic multifactorial inflammatory disease characterized by progressive destruction of the tooth-supporting tissues, including the gingiva, periodontal ligament, cementum, and alveolar bone. The disease results from a complex interaction between dysbiotic subgingival microbial communities and the host immune-inflammatory response. Although primarily localized to the oral cavity, periodontitis is increasingly recognized as a systemic inflammatory condition capable of influencing distant organs and physiological systems [3].

The ulcerated epithelium lining periodontal pockets provides a direct pathway for bacteria, endotoxins, and inflammatory mediators to enter the systemic circulation. Routine activities such as mastication, flossing, and toothbrushing may induce transient bacteremia, facilitating dissemination of periodontal pathogens and their virulence factors. Consequently, periodontitis is associated with elevated circulating levels of inflammatory biomarkers, including C-reactive protein (CRP), tumor necrosis factor-alpha (TNF-α), interleukin (IL)-1β, IL-6, and matrix metalloproteinases (MMPs), contributing to a persistent state of low-grade systemic inflammation [13].

Accumulating evidence has linked periodontitis to several chronic systemic conditions, including cardiovascular disease, diabetes mellitus, rheumatoid arthritis, chronic kidney disease, respiratory disorders, metabolic syndrome, and adverse pregnancy outcomes. Among these associations, the bidirectional relationship between periodontitis and diabetes mellitus is particularly well established, with periodontal inflammation contributing to impaired glycemic control and diabetes promoting periodontal tissue destruction. Notably, periodontal therapy has been shown to reduce systemic inflammatory burden and improve glycemic outcomes, highlighting the systemic consequences of periodontal disease [14].

The chronic inflammatory state associated with periodontitis may also have important implications for neurological health. Persistent exposure to inflammatory mediators and microbial products can influence central nervous system homeostasis through immune activation, vascular dysfunction, and disruption of blood-brain barrier integrity. Given the recognized role of neuroinflammation in neurodegenerative disease pathogenesis, periodontitis has emerged as a potential modifiable risk factor contributing to cognitive decline and neurodegeneration [3].

Mechanistic links between periodontitis and neurodegeneration

The association between periodontitis and neurodegenerative diseases is supported by multiple interconnected biological pathways involving chronic inflammation, microbial dissemination, blood-brain barrier (BBB) dysfunction, oxidative stress, immune dysregulation, and protein aggregation. Collectively, these mechanisms provide a biologically plausible framework linking oral dysbiosis with neurodegenerative pathology.

Neuroinflammation and Cytokine Cascade

Neuroinflammation is a central feature of neurodegenerative disorders, including Alzheimer’s disease (AD) and Parkinson’s disease (PD). Patients with periodontitis exhibit elevated systemic levels of pro-inflammatory mediators such as TNF-α, IL-1β, IL-6, IL-17, and interferon-gamma (IFN-γ), which can influence central nervous system (CNS) function directly or indirectly [5]. Persistent exposure to these mediators promotes microglial activation and sustained production of reactive oxygen species (ROS), nitric oxide, and inflammatory cytokines, creating a neurotoxic environment that contributes to neuronal injury and disease progression [2].

Blood-Brain Barrier Dysfunction

The BBB plays a critical role in maintaining CNS homeostasis by restricting the entry of pathogens and inflammatory molecules. Chronic systemic inflammation associated with periodontitis can disrupt endothelial tight junctions and increase BBB permeability [2]. In addition, lipopolysaccharides (LPS) derived from Gram-negative periodontal pathogens further enhance endothelial dysfunction. Compromised BBB integrity facilitates the entry of inflammatory mediators and microbial products into neural tissues, thereby amplifying neuroinflammatory responses [10].

Bacterial Invasion and Direct Neurotoxicity

Periodontal pathogens may access neural tissues through hematogenous dissemination or cranial nerve pathways. Among these microorganisms, *Porphyromonas gingivalis* has been most strongly implicated in neurodegeneration. DNA, LPS, and gingipain proteases derived from *P. gingivalis* have been identified in postmortem brain tissues of individuals with AD [4]. Experimental studies suggest that gingipains contribute to neuronal injury, synaptic dysfunction, amyloid-beta accumulation, and tau hyperphosphorylation, while pharmacological inhibition of these virulence factors has demonstrated neuroprotective effects in preclinical models [6].

Molecular Mimicry and Autoimmune Responses

Structural similarities between microbial antigens and host proteins may induce cross-reactive immune responses that contribute to neuronal injury. Chronic exposure to periodontal pathogens can stimulate adaptive immune responses and autoantibody production, potentially exacerbating neuroinflammation and neurodegeneration [15]. Although direct evidence remains limited, molecular mimicry represents a plausible mechanism linking chronic oral infection with neurological disease.

Oxidative Stress and Mitochondrial Dysfunction

Oxidative stress is a shared pathological feature of both periodontitis and neurodegenerative disorders. Excessive ROS production during periodontal inflammation may overwhelm antioxidant defenses, resulting in lipid peroxidation, DNA damage, and mitochondrial dysfunction [5]. Given the high metabolic demands of neurons, chronic oxidative stress can accelerate neuronal apoptosis and promote pathological protein aggregation [2].

Amyloidogenesis and Tau Pathology

Chronic infection and inflammation may influence hallmark neuropathological processes associated with AD. Experimental evidence indicates that *P. gingivalis* and its virulence factors promote amyloid-beta production and tau hyperphosphorylation [6]. While amyloid-beta may initially function as an antimicrobial peptide, persistent microbial stimulation can lead to excessive plaque formation, neurotoxicity, and neurofibrillary tangle development, thereby contributing to cognitive decline [4,6]. The principal biological mechanisms linking periodontitis and neurodegeneration through the oral-brain axis are summarized in Table 1.

**Table 1:** Biological mechanisms underlying the oral-brain axis. BBB: blood-brain barrier; GSK-3β: glycogen synthase kinase-3 beta; CDK5: cyclin-dependent kinase 5; IL: interleukin; LPS: lipopolysaccharide; TNF-α: tumor necrosis factor-alpha

Mechanism	Biological process	Potential neurological consequences
Systemic inflammation	Release of pro-inflammatory cytokines (TNF-α, IL-1β, IL-6) and acute-phase proteins such as CRP from periodontal tissues into systemic circulation	Neuroinflammation, microglial activation, synaptic dysfunction, and neuronal injury
Blood-brain barrier dysfunction	Cytokine-mediated disruption of endothelial tight junctions and increased BBB permeability	Enhanced entry of inflammatory mediators, immune cells, and microbial products into the central nervous system
Hematogenous dissemination	Dissemination of periodontal pathogens and their virulence factors (e.g., lipopolysaccharide, gingipains) through the bloodstream	Direct microbial effects on neural tissues and amplification of neuroinflammatory responses
Neural dissemination	Potential migration of pathogens or inflammatory signals via cranial nerves, particularly the trigeminal nerve	Local neuroinflammation and propagation of neuropathological changes
Oxidative stress	Excessive generation of reactive oxygen species, mitochondrial dysfunction, and impaired antioxidant defenses	Neuronal apoptosis, cellular damage, and promotion of protein misfolding
Molecular mimicry	Cross-reactive immune responses between bacterial antigens and host neural proteins	Immune-mediated neuronal injury and chronic neuroinflammation
Amyloidogenesis	Infection- and inflammation-induced amyloid-β production as part of the innate immune response	Amyloid plaque accumulation, neurotoxicity, and progression of neurodegeneration
Tau hyperphosphorylation	Activation of inflammatory signaling pathways and kinases (e.g., GSK-3β, CDK5) leading to abnormal tau phosphorylation	Neurofibrillary tangle formation and neuronal dysfunction
Oral-gut-brain axis interaction	Oral dysbiosis alters gut microbiota composition, intestinal permeability, and immune signaling	Enhanced systemic inflammation, neuroimmune dysregulation, and potential acceleration of neurodegenerative processes

Shared and distinct mechanisms in Alzheimer’s disease and Parkinson’s disease

Although Alzheimer’s disease (AD) and Parkinson’s disease (PD) are distinct neurodegenerative disorders with different clinical manifestations and neuropathological hallmarks, accumulating evidence suggests that they share several pathogenic mechanisms through which periodontitis may contribute to disease onset and progression. Chronic systemic inflammation, persistent activation of microglia, oxidative stress, blood-brain barrier dysfunction, and dissemination of periodontal pathogens and their virulence factors constitute common biological pathways implicated in both disorders [2-6,13-15]. In AD, these mechanisms have been associated with amyloid-β deposition, tau hyperphosphorylation, synaptic dysfunction, and progressive cognitive decline, with *Porphyromonas gingivalis* and its gingipain proteases providing substantial mechanistic support for the oral-brain axis hypothesis [4,6,7]. In contrast, PD is characterized predominantly by degeneration of dopaminergic neurons within the substantia nigra, where chronic neuroinflammation, immune dysregulation, and oxidative stress are believed to accelerate neuronal injury and disease progression [2,5]. Although direct mechanistic evidence is currently more robust for AD than for PD, both disorders appear to share upstream inflammatory and microbial pathways originating from periodontal dysbiosis [3-6,15]. Collectively, these observations support the oral-brain axis as a common biological framework linking chronic periodontal inflammation with multiple neurodegenerative disorders while emphasizing that the downstream neuropathological consequences are determined by disease-specific molecular and cellular vulnerabilities.

Oral-gut-brain axis interactions

Recent evidence has expanded the concept of the oral-brain axis to include interactions with the gut microbiome. Oral microorganisms can be swallowed and subsequently influence gastrointestinal microbial communities, leading to dysbiosis, intestinal inflammation, and increased gut permeability [16]. These alterations may facilitate systemic dissemination of microbial metabolites and inflammatory mediators, thereby contributing to chronic immune activation [17].

Through modulation of the gut-brain axis, oral dysbiosis may influence neuroimmune regulation, neuroinflammation, and neuronal homeostasis [17,18]. Although this pathway remains incompletely understood, growing evidence suggests that interconnected disturbances within oral and gut microbial ecosystems may contribute to the development and progression of neurodegenerative disorders [18].

**Figure 2:**
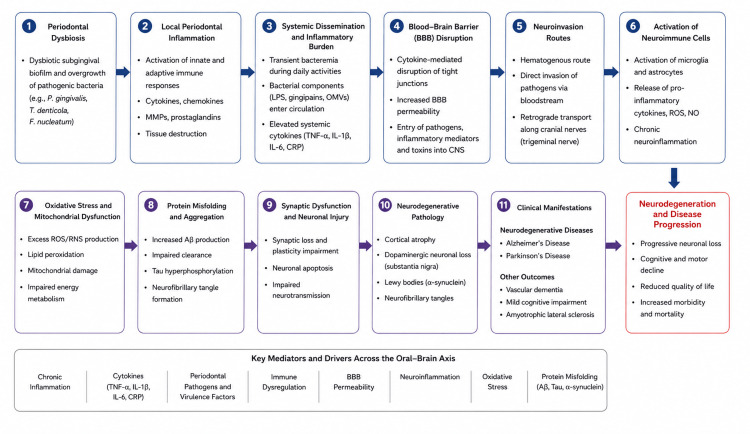
Mechanistic pathways of the oral-brain axis (from periodontal dysbiosis to neurodegeneration). Proposed sequence of biological events linking periodontal dysbiosis with neurodegenerative disease progression. Oral dysbiosis initiates local periodontal inflammation, resulting in systemic dissemination of pathogens, virulence factors, and inflammatory mediators. Subsequent blood-brain barrier dysfunction, neuroimmune activation, oxidative stress, protein misfolding, synaptic dysfunction, and neuronal loss contribute to the development and progression of Alzheimer's disease, Parkinson's disease, and related neurodegenerative disorders. This image was created by the authors of this study using Microsoft PowerPoint 2021 software (Redmond, WA: Microsoft Corp.).

Collectively, these interconnected pathways establish a biologically plausible framework that links periodontal dysbiosis to neurodegeneration through systemic inflammation, microbial dissemination, blood-brain barrier dysfunction, neuroimmune activation, oxidative stress, protein aggregation, and neuronal injury. The principal mechanistic pathways underlying the oral-brain axis are summarized in Figure 2.

Periodontal pathogens implicated in neurodegeneration

Although periodontitis is a polymicrobial disease, several periodontal pathogens have been implicated in neurodegenerative processes through their ability to induce chronic inflammation, evade host defenses, and disseminate beyond the oral cavity. Among these microorganisms, *Porphyromonas gingivalis *has received the greatest attention because of its strong association with Alzheimer's disease (AD) and its capacity to influence multiple neuropathological pathways [19].

*Porphyromonas gingivalis* is considered a keystone periodontal pathogen capable of driving microbial dysbiosis and chronic inflammation. Detection of bacterial DNA, lipopolysaccharides (LPS), and gingipain proteases in postmortem brain tissues has provided evidence supporting its potential involvement in AD pathogenesis [4]. Experimental studies have demonstrated that *P. gingivalis* and its virulence factors promote neuroinflammation, amyloid-beta accumulation, tau hyperphosphorylation, synaptic dysfunction, and cognitive impairment [6].

*Treponema denticola* is a highly invasive spirochete frequently detected in advanced periodontal lesions. Oral spirochetes have been identified in brain tissues of patients with AD, suggesting a possible role in neuroinflammatory responses and neuronal injury [20]. Its invasive properties and ability to evade host immune defenses may facilitate dissemination beyond the oral cavity.

*Aggregatibacter actinomycetemcomitans* produces leukotoxin A and other virulence factors capable of inducing robust inflammatory responses. Although direct evidence linking this organism to neurodegenerative disease remains limited, chronic systemic inflammation associated with infection may contribute indirectly to neuronal dysfunction and disease progression [15].

*Fusobacterium nucleatum* possesses strong adhesive and invasive properties that facilitate systemic dissemination and inflammatory activation [15]. While its role in neurodegeneration remains incompletely understood, its ability to promote vascular dysfunction and chronic inflammation suggests a potential contribution to neurological pathology.

In addition to these organisms, pathogens such as *Tannerella forsythia, Prevotella intermedia, and Campylobacter rectus* may contribute to neurodegenerative processes through persistent immune activation, oxidative stress, and systemic inflammatory signaling [15]. Collectively, these findings highlight the importance of oral microbial dysbiosis as a potential contributor to the oral-brain axis and neurodegenerative disease pathogenesis. The major periodontal pathogens implicated in neurodegeneration and their proposed neuropathogenic effects are summarized in Table 2.

**Table 2:** Periodontal pathogens implicated in neurodegenerative diseases and their proposed neuropathogenic effects. CDT: cytolethal distending toxin; LPS: lipopolysaccharide

Pathogen	Major virulence factors	Proposed neurological effects
Porphyromonas gingivalis	Gingipains, lipopolysaccharide (LPS), fimbriae, outer membrane vesicles	Neuroinflammation, microglial activation, amyloid-β accumulation, tau hyperphosphorylation, neuronal injury
Treponema denticola	Dentilisin, major sheath proteins, motility-associated factors	Tissue invasion, neuroinflammatory activation, blood-brain barrier disruption, neuronal damage
Tannerella forsythia	BspA protein, proteolytic enzymes, surface layer proteins	Chronic inflammation, immune dysregulation, and potential contribution to neuroinflammatory pathways
Aggregatibacter actinomycetemcomitans	Leukotoxin A, cytolethal distending toxin (CDT)	Systemic inflammation, immune activation, and potential indirect neuronal injury
Fusobacterium nucleatum	FadA adhesin, endotoxins, outer membrane proteins	Enhanced inflammatory signaling, endothelial dysfunction, and potential blood-brain barrier impairment
Prevotella intermedia	Proteases, LPS, hemolysins	Chronic immune activation, oxidative stress, and amplification of systemic inflammation
Campylobacter rectus	Cytotoxins, endotoxins, surface-layer proteins	Systemic inflammatory burden, endothelial dysfunction, and indirect neuroinflammatory effects

Evidence from animal models and experimental studies

Experimental and preclinical studies have provided important mechanistic evidence supporting the role of the oral-brain axis in neurodegenerative disease pathogenesis. Animal models and in vitro investigations have demonstrated that chronic periodontal infection can induce neuroinflammation, neuronal injury, and neuropathological changes characteristic of Alzheimer's disease (AD) and other neurodegenerative disorders [7].

Murine studies have shown that oral infection with *Porphyromonas gingivalis* results in bacterial colonization of brain tissues, activation of microglia, increased production of pro-inflammatory cytokines, and accumulation of amyloid-beta plaques [7]. These findings support the hypothesis that chronic periodontal infection may directly contribute to neurodegenerative processes through microbial dissemination and sustained inflammatory signaling.

Cell culture studies further demonstrate the neurotoxic potential of periodontal pathogens and their virulence factors. Gingipains, the major proteases produced by *P. gingivalis*, have been shown to induce neuronal injury, synaptic dysfunction, and activation of inflammatory pathways. Conversely, pharmacological inhibition of gingipains has been associated with reduced neuroinflammation and improved neuronal survival, highlighting their potential as therapeutic targets [6].

Additional experimental evidence indicates that administration of periodontal pathogen-derived lipopolysaccharides (LPS) can induce cognitive impairment, oxidative stress, and neuroinflammatory responses resembling those observed in human neurodegenerative diseases [21]. Similarly, transgenic AD mouse models exposed to chronic periodontal infection exhibit increased amyloid-beta deposition, enhanced tau pathology, and worsened cognitive performance compared with non-infected controls [6,7].

Although animal and laboratory models cannot fully replicate the complexity of human neurodegenerative disorders, the consistency of findings across experimental systems provides strong biological support for the oral-brain axis. Collectively, these studies reinforce the concept that periodontal pathogens and chronic oral inflammation may contribute to neurodegeneration through interconnected microbial, inflammatory, and neuroimmune mechanisms.

Clinical and epidemiological evidence

An expanding body of epidemiological evidence supports an association between periodontitis and neurodegenerative disorders, particularly Alzheimer's disease (AD), Parkinson's disease (PD), cognitive impairment, and dementia [5,8,9,15,22-24]. Observational studies conducted across diverse populations suggest that chronic periodontal inflammation may contribute to neurodegenerative risk through interconnected microbial and inflammatory pathways [8-10,15,22].

Longitudinal and population-based cohort studies provide important evidence supporting a temporal relationship between periodontal disease and neurodegeneration. In a large retrospective cohort study from Taiwan, Chen et al. reported an increased risk of AD among individuals with chronic periodontitis, particularly those with prolonged disease duration [8]. Similarly, Ide et al. demonstrated that periodontitis was associated with accelerated cognitive decline in patients with established AD, suggesting a potential role in disease progression [5].

Evidence linking periodontal disease and PD has also strengthened in recent years. Jeong et al., in a nationwide South Korean cohort, identified periodontitis as an independent risk factor for PD after adjustment for major confounding variables [9]. Furthermore, population-based studies have reported that regular dental scaling and periodontal care are associated with a reduced risk of PD, highlighting the potential importance of oral health maintenance in neurological disease prevention [25].

Several investigations have also demonstrated associations between periodontal parameters and cognitive performance. Measures such as periodontal pocket depth, clinical attachment loss, tooth loss, and elevated serum antibody responses to periodontal pathogens have been linked to poorer cognitive outcomes and increased dementia risk [5,10,15,22]. These findings support the hypothesis that chronic periodontal infection may influence neurodegenerative processes through sustained inflammatory and immune-mediated mechanisms [5,10,15].

Systematic reviews and meta-analyses further reinforce these observations. Collectively, available evidence indicates that periodontitis is associated with an increased risk of cognitive impairment, dementia, and AD, although the magnitude of association varies across studies [23,24]. Shared elevations in inflammatory mediators, including TNF-α, IL-1β, IL-6, and C-reactive protein (CRP), provide additional biological plausibility for this relationship [5,10].

Despite the consistency of these findings, important limitations remain. Most available studies are observational and therefore susceptible to residual confounding from factors such as aging, smoking, diabetes mellitus, socioeconomic status, education, and healthcare access [8,9,22-24]. Variations in periodontal diagnostic criteria and neurocognitive assessments further complicate comparisons across studies [23,24]. Consequently, while current evidence strongly supports an association between periodontal disease and neurodegenerative disorders, definitive causal relationships remain to be established through prospective longitudinal studies and randomized interventional trials [23,24]. A summary of representative epidemiological studies investigating the association between periodontitis and neurodegenerative diseases is presented in Table 3.

**Table 3:** Major epidemiological studies investigating the association between periodontitis and neurodegenerative diseases through the oral-brain axis. Studies consistently demonstrate associations between periodontal inflammation, cognitive decline, dementia, Alzheimer's disease, and Parkinson's disease, supporting a potential role of oral health as a modifiable factor in neurodegenerative disease risk. AD: Alzheimer's disease; PD: Parkinson's disease; TNF-α: tumor necrosis factor-alpha

Studies	Country	Design	Sample size	Outcome	Key findings
Chen et al. (2017) [8]	Taiwan	Retrospective matched cohort study (National Health Insurance Research Database)	9,291 patients with chronic periodontitis and 18,672 controls	Alzheimer's disease	Individuals with chronic periodontitis who had ≥10 years of exposure exhibited a significantly higher risk of developing Alzheimer's disease (adjusted HR=1.707, 95% CI: 1.152-2.528). The findings suggest a long-term association between chronic periodontitis and AD risk.
Jeong et al. (2021) [9]	South Korea	Nationwide population-based cohort study	153,165 participants	Parkinson's disease	Periodontitis was associated with a modest but statistically significant increase in the risk of incident Parkinson's disease. Participants with periodontitis requiring further dental treatment had a higher risk of Parkinson's disease (HR: 1.142, 95% CI: 1.094-1.193), while the highest risk was observed among individuals with both periodontitis and metabolic syndrome (HR: 1.167, 95% CI: 1.118-1.219).
Ide et al. (2016) [5]	United Kingdom	Prospective longitudinal cohort study	60 patients with Alzheimer's disease	Cognitive decline in Alzheimer's disease	Baseline periodontitis was associated with a six-fold greater rate of cognitive decline over a 6-month follow-up period, independent of baseline cognitive status. Periodontitis was also associated with increased systemic inflammatory markers, suggesting a potential inflammatory mechanism linking periodontal disease and cognitive deterioration.
Noble et al. (2014) [15]	United States	Case-cohort study nested within a longitudinal cohort	219 participants (110 incident AD cases, 109 controls)	Incident Alzheimer's disease	Elevated serum IgG antibody levels against periodontal microbiota were associated with the risk of developing Alzheimer's disease. High anti-*Actinomyces naeslundii* titers were linked to an increased risk of incident AD (HR=2.0; adjusted HR=3.1), whereas high anti-*Eubacterium nodatum* titers were associated with a reduced risk (HR=0.5).
Lee et al. (2020) [22]	Taiwan	Nationwide retrospective cohort study	112,036 participants (56,018 with periodontitis; 56,018 matched controls)	Dementia	Periodontitis was associated with a significantly increased risk of dementia (HR=1.79, 95% CI: 1.67-1.93). The association was observed in both sexes and was particularly evident among individuals aged >60 years. Among patients with periodontitis, statin use, metformin use, and influenza vaccination were associated with a reduced risk of dementia.
Chen et al. (2018) [25]	Taiwan	Nationwide population-based nested case-control study	23,825 participants (4,765 Parkinson's disease cases; 19,060 controls)	Parkinson's disease	Regular dental scaling was associated with a reduced risk of Parkinson's disease. Among individuals aged 40-69 years without periodontal inflammatory disease, dental scaling performed for five consecutive years was associated with a significantly lower risk of PD (adjusted OR=0.204, 95% CI: 0.047-0.886). The findings suggest that long-term preventive periodontal care may protect against Parkinson's disease development.
Kaliamoorthy et al. (2022) [23]	Multiple Countries	Systematic review and meta-analysis	5 studies in qualitative review; 3 studies in meta-analysis	Alzheimer's disease	Meta-analysis demonstrated a significant association between periodontitis and Alzheimer's disease, with individuals with periodontitis showing a higher likelihood of AD (OR=1.67, 95% CI: 1.21-2.32).
Kamer et al. (2009) [10]	United States	Case-control study	34 participants (18 Alzheimer's disease patients, 16 cognitively normal controls)	Alzheimer's disease	Plasma TNF-α levels and serum IgG antibodies against periodontal bacteria were significantly elevated in Alzheimer's disease patients compared with controls. The combination of systemic inflammatory markers and periodontal bacterial antibodies improved discrimination between AD patients and cognitively normal individuals, supporting a role for periodontal infection-associated inflammation in AD.
Leira et al. (2017) [24]	Multiple Countries	Systematic review and meta-analysis	5 observational studies (2 cross-sectional, 2 case-control, 1 cohort)	Alzheimer's disease	Meta-analysis demonstrated a significant association between periodontal disease and Alzheimer's disease (OR=1.69, 95% CI: 1.21-2.35). Severe periodontal disease showed an even stronger association with AD (OR=2.98, 95% CI: 1.58-5.62).

Collectively, evidence supporting the oral-brain axis derives from complementary preclinical, observational, and clinical investigations, each contributing distinct strengths while presenting important limitations. Experimental studies provide strong mechanistic evidence demonstrating that periodontal pathogens, particularly *Porphyromonas gingivalis*, can induce neuroinflammation, amyloid-β deposition, tau hyperphosphorylation, and neuronal injury under controlled conditions. However, these models may not fully replicate the complexity of human neurodegenerative diseases. Observational epidemiological studies consistently report associations between periodontitis and Alzheimer’s disease, Parkinson’s disease, and cognitive decline, often across large populations and long follow-up periods. Nevertheless, such studies remain vulnerable to residual confounding, reverse causation, and variability in periodontal and neurological diagnostic criteria. Clinical evidence remains comparatively limited, with few prospective longitudinal cohorts and almost no randomized interventional trials evaluating whether periodontal treatment alters neurodegenerative outcomes. Consequently, while the cumulative evidence supports biological plausibility, definitive causal relationships remain to be established [6].

Therapeutic and preventive implications

Recognition of the oral-brain axis has important implications for the prevention and management of neurodegenerative diseases. If periodontal disease contributes to neurodegeneration through chronic inflammation, microbial dissemination, and neuroimmune dysregulation, maintaining periodontal health may be a practical, potentially modifiable strategy for reducing neurological risk [5,8,22].

Conventional periodontal therapies effectively reduce microbial burden and local inflammation and have been associated with improvements in systemic inflammatory profiles, including reductions in circulating levels of CRP, IL-6, and TNF-α [14,26]. Such effects may have broader implications for mitigating neuroinflammatory processes implicated in cognitive decline and neurodegenerative disease progression [5,22].

Beyond conventional treatment, emerging approaches aimed at restoring microbial homeostasis and modulating host inflammatory responses may offer additional benefits. Advances in microbiome-targeted therapies, host-modulation strategies, precision oral healthcare, and pathogen-specific interventions have generated interest in their potential to disrupt the biological pathways linking periodontal disease and neurodegeneration [6,19].

The growing recognition of oral health as a component of systemic and neurological well-being highlights the need for interdisciplinary collaboration among dental professionals, neurologists, geriatricians, and primary care physicians. Incorporating periodontal assessment and preventive oral healthcare into healthy aging programs may contribute to comprehensive strategies aimed at preserving cognitive and neurological function [5,8].

Despite promising evidence, definitive clinical benefits remain to be established. Future research should prioritize large-scale prospective studies and randomized controlled trials to determine whether periodontal interventions can reduce the incidence, delay the onset, or slow the progression of neurodegenerative diseases. Identification of reliable biomarkers and therapeutic targets within the oral-brain axis may further facilitate the development of preventive and personalized treatment strategies [6,19]. The principal therapeutic strategies targeting the oral-brain axis are summarized in Table 4.

**Table 4:** Therapeutic strategies targeting the oral-brain axis.

Intervention	Primary target	Potential neurological benefit
Scaling and root planing	Reduction of periodontal biofilm, bacterial load, and local inflammation	Reduced systemic inflammatory burden and potential attenuation of neuroinflammatory processes
Periodontal maintenance therapy	Prevention of periodontal disease recurrence and sustained periodontal health	Long-term reduction of systemic inflammatory mediators and microbial burden
Antimicrobial therapy	Suppression of pathogenic oral microbiota	Reduced microbial dissemination and systemic inflammatory activation
Host-modulation therapy	Regulation of excessive inflammatory responses	Potential attenuation of neuroinflammatory signaling pathways
Probiotics and prebiotics	Restoration of oral and gut microbial homeostasis	Improved oral-gut microbiome balance and modulation of immune responses
Anti-inflammatory agents	Inhibition of cytokine-mediated inflammation	Potential neuroprotective effects through reduction of chronic inflammation
Gingipain inhibitors	Targeting *Porphyromonas gingivalis* virulence factors	Potential reduction of amyloidogenic, neuroinflammatory, and neurotoxic processes
Precision oral healthcare	Personalized risk assessment, prevention, and monitoring	Early identification and management of individuals at increased risk of neurodegenerative disorders

Current challenges and knowledge gaps

Despite growing evidence supporting the oral-brain axis, several important limitations remain. Most available clinical studies are observational and therefore cannot establish definitive causal relationships between periodontitis and neurodegenerative diseases. Reverse causality also remains a concern, as cognitive impairment and motor dysfunction may compromise oral hygiene practices and increase susceptibility to periodontal disease.

Considerable heterogeneity exists across studies with respect to periodontal disease definitions, diagnostic criteria, cognitive assessments, and adjustment for confounding variables. Factors such as aging, smoking, diabetes mellitus, socioeconomic status, nutrition, and healthcare access may independently influence both periodontal and neurological outcomes, complicating interpretation of epidemiological findings.

Although experimental studies provide compelling mechanistic evidence, translation of findings from animal models to human disease remains challenging. Furthermore, the complex interplay among host genetics, immune responses, microbial composition, and environmental influences is not yet fully understood. Addressing these limitations will require standardized methodologies, longitudinal investigations, and interdisciplinary research approaches capable of clarifying the causal significance of the oral-brain axis.

Future perspectives

Future research should focus on elucidating the molecular and cellular mechanisms through which oral dysbiosis influences neurodegenerative processes. Advances in multi-omics technologies, including metagenomics, transcriptomics, proteomics, and metabolomics, offer unprecedented opportunities to characterize host-microbe interactions and identify biomarkers associated with neurological risk.

Salivary diagnostics represent a particularly promising area of investigation because saliva contains microbial, inflammatory, and genetic signatures that may facilitate early detection of both periodontal and neurodegenerative diseases. Integration of artificial intelligence and machine-learning approaches with biomarker profiling may further improve risk prediction and support the development of personalized preventive strategies.

Emerging therapeutic approaches targeting microbial dysbiosis, neuroinflammation, immune modulation, and blood-brain barrier preservation may provide novel opportunities for intervention along the oral-brain axis. In parallel, well-designed longitudinal cohorts and randomized clinical trials are needed to determine whether periodontal interventions can modify neurodegenerative disease risk or progression. As evidence continues to evolve, oral healthcare may become an increasingly important component of multidisciplinary strategies aimed at promoting healthy aging, preserving cognitive function, and reducing the burden of neurodegenerative diseases.

## Conclusions

The oral-brain axis has emerged as a promising conceptual framework linking periodontitis with neurodegenerative diseases through interconnected microbial, inflammatory, immune, and neurovascular mechanisms. Current evidence from experimental, epidemiological, and clinical studies supports a biologically plausible association between periodontal disease and both Alzheimer’s disease and Parkinson’s disease, with chronic oral dysbiosis potentially contributing to neuroinflammation, blood-brain barrier dysfunction, oxidative stress, and pathological protein aggregation. However, the existing evidence is predominantly observational and preclinical, precluding definitive conclusions regarding causality.

Several important challenges remain unresolved. It is unclear whether periodontitis functions as an independent risk factor, an accelerator of neurodegenerative processes, or a surrogate marker of systemic inflammation. Moreover, although *Porphyromonas gingivalis* has been extensively investigated, neurodegeneration is likely influenced by complex interactions within the broader oral microbiome rather than by a single pathogen. Variability in periodontal diagnostic criteria, cognitive assessment methods, and control of confounding factors further limits the comparability of existing studies. Future research should prioritize standardized longitudinal cohort studies and well-designed randomized clinical trials to determine whether periodontal interventions can modify neuroinflammatory biomarkers, delay cognitive decline, or influence the progression of neurodegenerative diseases. Integration of multi-omics technologies, microbiome profiling, and biomarker-based approaches may further elucidate the molecular mechanisms underlying the oral-brain axis and facilitate the identification of novel therapeutic targets. Although causality remains to be established, maintaining periodontal health represents a practical and potentially modifiable component of strategies aimed at promoting healthy aging and preserving neurological function. A more comprehensive understanding of the oral-brain axis may ultimately support the development of integrated preventive and therapeutic approaches that reduce the burden of neurodegenerative diseases.
